# A Practical Method for Blind Pixel Detection for the Push-Broom Thermal-Infrared Hyperspectral Imager

**DOI:** 10.3390/s22197403

**Published:** 2022-09-29

**Authors:** Bingxin Liu, Yulong Du, Chengyu Liu, Ying Li

**Affiliations:** 1 Navigation College, Dalian Maritime University, Dalian 116026, China; 2 Key Laboratory of Space Active Opto-Electronic Technologies, Shanghai Institute of Technical Physics, Chinese Academy of Sciences, Shanghai 200083, China

**Keywords:** thermal infrared, hyperspectral imager, blind pixel

## Abstract

Thermal infrared hyperspectral imager is one of the frontier payloads in current hyperspectral remote sensing research. It has broad application prospects in land and ocean temperature inversion, environmental monitoring, and other fields. However, due to the influence of the production process of the infrared focal plane array and the characteristics of the material itself, the infrared focal plane array inevitably has blind pixels, resulting in spectral distortion of the data or even invalid data, which limits the application of thermal infrared hyperspectral data. Most of the current blind pixels detection methods are based on the spatial dimension of the image, that is, processing single-band area images. The push-broom thermal infrared hyperspectral imager works completely different from the conventional area array thermal imager, and only one row of data is obtained per scan. Therefore, the current method cannot be directly applied to blind pixels detection of push-broom thermal infrared hyperspectral imagers. Based on the imaging principle of push-broom thermal infrared hyperspectral imager, we propose a practical blind pixels detection method. The method consists of two stages to detect and repair four common types of blind pixels: dead pixel, dark current pixel, blinking pixel, and noise pixel. In the first stage, dead pixels and dark current pixels with a low spectral response rate are detected by spectral filter detection; noise pixels are detected by spatial noise detection; and dark current pixels with a negative response slope are detected by response slope detection. In the second stage, according to the random appearance of blinking pixels, spectral filter detection is used to detect and repair spectral anomalies caused by blinking pixels line by line. In order to verify the effectiveness of the proposed method, a flight test was carried out, using the Airborne Thermal-infrared Hyperspectral Imaging System (ATHIS), the latest thermal infrared imager in China, for data acquisition. The results show that the method proposed in this paper can accurately detect and repair blind pixel, thus effectively eliminating spectral anomalies and significantly improving image quality.

## 1. Introduction

Hyperspectral thermal infrared data contains rich spectral information that can reveal the radiation changes in detail and reflect the unique diagnostic characteristics in the thermal infrared spectrum. It provides more reasonable assumptions and constraints and has important research value and application prospects [1]. Hyperspectral thermal infrared remote sensing can be widely used in geological mapping, resource exploration, surface temperature detection, urban heat flow analysis, environmental monitoring, land object classification, and other fields, and it has become an important research direction and a breakthrough point in the field of thermal infrared remote sensing [2,3].

For the development of hyperspectral thermal infrared sensors, due to the limitations of key technologies, such as area array infrared focal planes, low-temperature optical systems, and fine spectroscopy, the current spaceborne hyperspectral thermal infrared sensors generally use non-imaging modes. In contrast, airborne hyperspectral thermal infrared sensors can overcome these limitations, and imagers can be used to simultaneously acquire image and spectral data [1]. However, due to the influence of the production process of the infrared focal plane array and the characteristics of the material itself, the infrared focal plane array inevitably has blind pixels, resulting in spectral distortion of the data or even invalid data, which seriously affects the image quality and subsequent application of data [4,5].

According to the response characteristics, blind pixel can be divided into four types: (1) Dead pixel, which has no response to the input radiation, and always outputs a constant value; (2) Noise pixel, which contains extremely noisy and fluctuating value; (3) Dark current pixel, which appears white in a dark environment, but behaves normally in a bright environment; and (4) Blinking pixel, which has the characteristics of random flickering. In a certain period of time, the output level of the blinking pixel fluctuates greatly, and sometimes it tends to be normal. It is difficult to distinguish it from normal pixels in the average value of multiple frames, but its standard difference is greater than normal pixels.

In terms of blind pixel detection, there are currently two main methods, the calibration method and the scene-based method. The calibration method involves obtaining images of uniform incident radiation by imaging a reference source, and using difference detection or sequence statistics for blind pixel detection [6,7]. In the sequence image statistical method, the pixel responsibility and noise at different temperatures are calculated first, and the value of each pixel is compared with the average responsibility or noise voltage. Those over 10 times the average or less than 1/10 of the average are considered as blind pixels [6]. Li [7] proposed a temporary mean outlier extraction (TMOE) detection algorithm for the characteristics that the gray value of blind pixels is basically fixed and abnormal. In the repair of blind pixels, an algorithm based on correlated pixel weighted interpolation is proposed. Li et al. [8] proposed an improved TMOE algorithm, which obtains the time-domain average background image from continuous infrared images and uses the threshold to extract blind pixels. The scene based method detects the position of blind pixels directly from the actual imaging data according to the difference between the response characteristics of blind pixels and normal pixels and applies the two-dimensional space method of windowing template. Zhang et al. [9] eliminated the influence of band noise on blind pixel detection by using the multi-directionality of dual density dual tree complex wavelet and generalized Gaussian distribution based on uniform background, and then used 3σ Criterion for blind pixel detection. Huang et al. [10] proposed a blind pixel detection method based on dynamic scenes. The bright and dark spots in the image are used as targets, and the blind pixels are detected by an iteratively modified background prediction model. Zheng et al. [11] proposed a blind pixel detection method based on small sliding window. On the basis of non-uniform correction and edge filtering, the mean and median values as well as the first-order gradient were calculated. Gradient weighted calculation of pixels were carried out. Finally, the weighting calculation was performed, and the threshold was set to compare with the original pixel for blind pixel detection. Currently, blind pixel detection is mostly based on the spatial dimension, which is suitable for a single-band area array image.

However, the current method cannot be directly applied to blind pixel detection of push-broom thermal infrared hyperspectral imagers. The push-broom thermal infrared hyperspectral imager works completely different from the conventional area array thermal imager. For the conventional area-array thermal imager, each frame is an image with a dimension of m × n (m, n are the number of lines and samples of the image, and both are greater than 1). On the data obtained at different times, pixels at the same location are collected by the same detector. Therefore, on the image of the conventional area array thermal imager, the influence of the blind pixel extends in the time dimension but appears as a random pattern in the spatial dimension. In contrast, the push-broom thermal infrared hyperspectral imager obtains an image with a dimension of 1 × n × z (1 line, n samples, z bands) per scan. The data at the same sample comes from the same detector. Therefore, on thermal infrared hyperspectral images, the data corruption caused by blind pixel has obvious features distributed across each line at the same sample position, and appears as randomness in the spectrum. Therefore, it is difficult to use the current method to detect blind pixel of push-broom thermal infrared hyperspectral imager.

At present, there are few published studies on blind pixel detection for push-broom thermal infrared imagers. Zhang [12] has used spectral angle matching to achieve blind pixel detection. However, this method requires black body data at more than three different temperatures. It is only suitable during comprehensive instrument testing in the laboratory, but it is not applicable when obtaining data from field flight. Herein, we proposed a blind pixel detection method for push-broom thermal infrared hyperspectral imagers and applied it to the data acquired by ATHIS. Results on laboratory and on-board data show that the proposed method could accurately detect and repair blind pixel, thus effectively eliminating spectral anomalies and significantly improving the image quality.

## 2. Data and Materials

### 2.1. ATHIS

**Figure 1:**
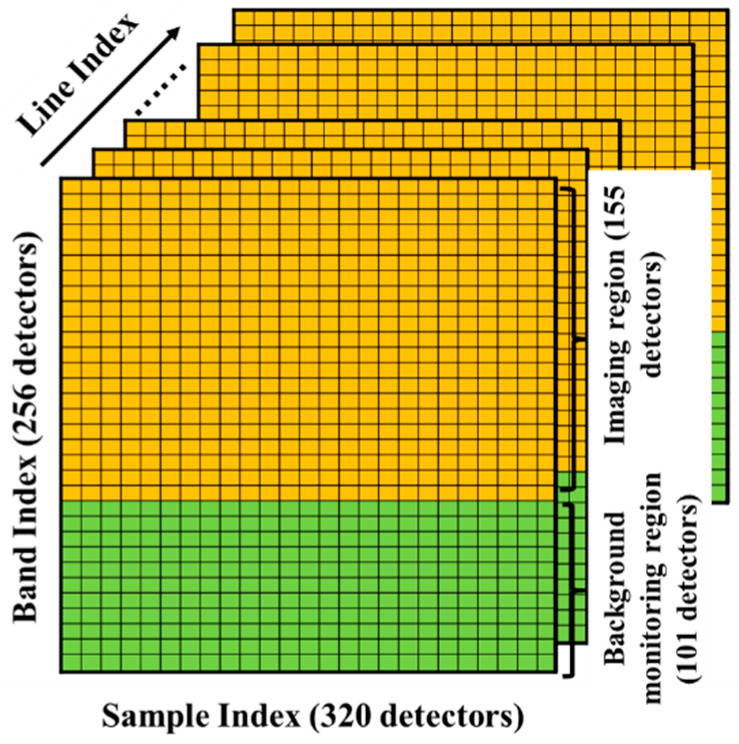
Schematic diagram of ATHIS data cube.

The ATHIS is a push-broom thermal infrared hyperspectral imager. The instrument is designed with 155 spectral bands, the spectral resolution is 38 nm, and the plane grating is used for spectroscopy. The designed spectral response range is 8.0–12.5 μm, and the detection sensitivity is better than 0.17 K @300 K on average. ATHIS has a field of view angle of 40° and a spatial resolution of 2 mrad. The size of the detector area array used by ATHIS is 320 × 256. As shown in Figure 1, ATHIS does not use all pixels as the imaging area. In the spectral dimension, some pixels are used to monitor the dark background. This design can largely eliminate the influence of the overall drift of the pixel value caused by the dark background during the operation.

### 2.2. Data Acquisition

The data obtained from the laboratory and the flight were used to verify the proposed blind pixel detection and repair method, respectively. In the laboratory, we use Ces200-06 low-275 black body as the radiation source. By adjusting the temperature of the black body, images of black body at different temperatures can be obtained. The size of the black body is 20 × 20 cm, which can cover the ATHIS field of view. The non-uniformity is better than ±0.0 °C (@23 °C), the temperature measurement error is ±0.15%, and the black body stability is ±0.05% within 30 min.

**Figure 2:**
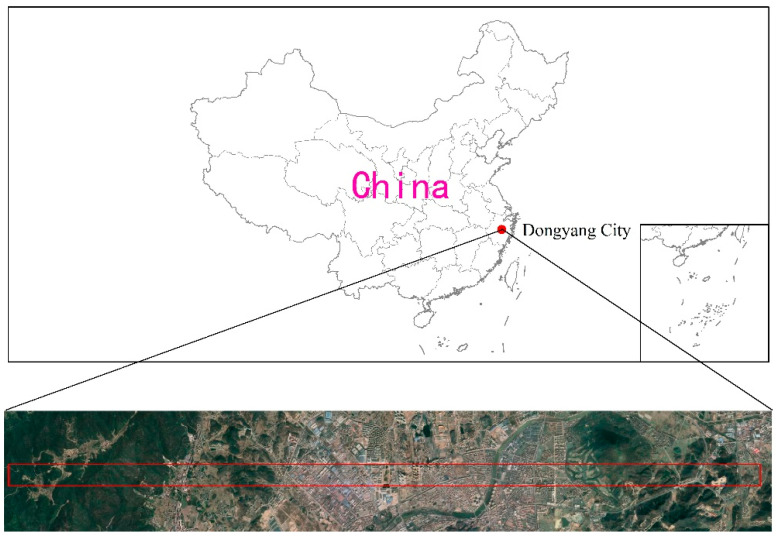
Location of the flight test.

Most dead pixels and dark current pixels could be detected in the laboratory because their positions are relatively stable. However, the location of the blinking pixel is changeable and needed to be detected in flight. In this regard, we conducted blind pixel detection during flight. The flight test was carried out in Dongyang City, Zhejiang Province, China (Figure 2). The relative altitude of the aviation experiment was 2000 m, and the corresponding ground spatial resolution was 2 m. The ATHIS instrument is placed on a specific gyro-stabilized platform (Leica Geosystems PAV80) and is simultaneously equipped with a high-precision IMU (PosPac 610 Applanix). A real-time calibration black body is installed under the platform for the radiation calibration of the instrument during flight.

## 3. Proposed Approach

**Figure 3:**
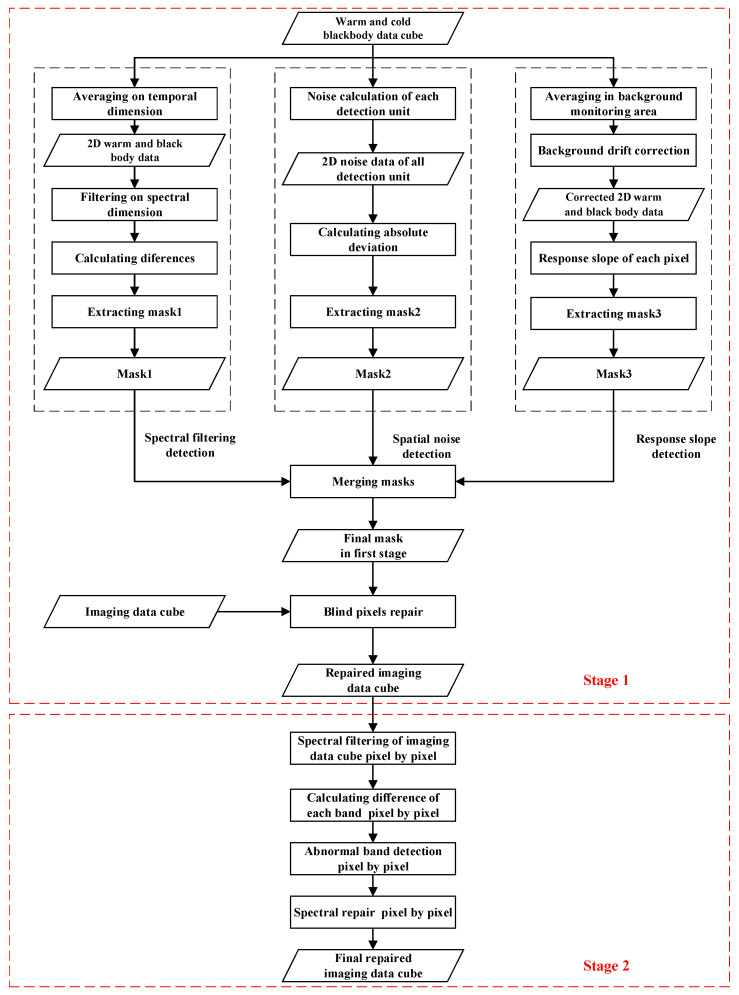
Technical flow chart.

As shown in Figure 3, there are two stages for blind pixel detection and repair. In the first stage, the detection of pixels of dead, noise, and dark current is performed based on the data of ATHIS calibration black body. The mask of blind pixels is obtained and used to repair the ATHIS images. The data of warm and cold black body are generally acquired in the gap of flight switching to achieve full optical path calibration. In the second stage, the abnormal spectra caused by the blinking pixel in the hyperspectral data cube are repaired, which is mainly based on the spectral dimension filtering.

### 3.1. Spectral Filtering Detection

As shown in Figure 1, for a frame of data cube acquired by ATHIS, the line direction of the ATHIS focal plane is the spatial dimension, and the direction along band index is the spectral dimension. The process of spectral filtering detection is divided into the following steps.

(1) On the temporal dimension of the obtained warm and cold black body data cubes, the average values are calculated, respectively.
  (1) $$DN_{bb}\left(i,k\right)=\frac{1}{N_{L,bb}}\sum_{j=1}^{N_{L,bb}}DN_{bb}\left(i,j,k\right)$$
  where, $DN_{bb}\left(i,j,k\right)$ is the data cube of calibration black body, the values of bb are cbb and wbb, which denote the warm and cold black body, respectively. *I*, *j* and *k* mean the *i-th* column, *j-th* row and *k-th* band. $DN_{bb}\left(i,k\right)$ is the 2D data of calibration black body after the average on the temporal dimension.
(2) Spectral filtering was performed on $DN_{bb}\left(i,k\right)$. A combination of median filtering and Savitzky–Golay (SG) filtering was used. This operation could avoid the influence of a large abnormal value for the SG filter and obtain a more accurate trend line. The calculation method is
  (2) $$DN_{bb,filtered}\left(i,\cdot \right)=SG\left\{median\left[DN_{bb}\left(i,\cdot \right)\right]\right\}$$
  where $DN_{bb,filtered}\left(i,\cdot \right)$ is the data after spectral filtering, and $median\left(\cdot \right)$ and $SG\left(\cdot \right)$ are the median and SG filter functions. The window size of the median filter and the SG filter was set to 5 for the best effect after many tests. The polynomial degree of SG filtering was set to 2.
(3) Then, the difference between $DN_{bb}\left(i,k\right)$ and $DN_{bb,filtered}\left(i,k\right)$ was calculated. $DN_{bb}\left(i,k\right)$ and $DN_{bb,filtered}\left(i,k\right)$ were the data sets of the black body before and after filtering, respectively. The difference is calculated as follows:
  (3) $$DN_{bb,diff}\left(i,k\right)=DN_{bb}\left(i,k\right)-DN_{bb,filtered}\left(i,k\right)$$
(4) The criterion of 3σ was used to extract a blind pixel mask on the $DN_{bb,diff}\left(i,k\right)$ column by column. The mask was noted as $mask_{1}\left(i,k\right)$.
  (4) $$mask_{1}\left(i,k\right)=\left\{\begin{array}{c} 1\;\;\;\;DN_{bb,diff}\left(i,k\right)>3\sigma_{bb}\left(i\right)\\ 0\;\;\;\;DN_{bb,diff}\left(i,k\right)\leq 3\sigma_{bb}\left(i\right) \end{array}\right.$$
  where $\sigma_{bb}\left(i\right)$ is the root mean square of the i-th column of the black body data. $\sigma \left(i\right)$ could be calculated as follows:
  (5) $$\sigma_{bb}\left(i\right)=\sqrt{\frac{1}{N_{B}}\sum_{k=1}^{N_{B}}DN_{bb,diff}^{2}\left(i,k\right)}$$
  where $N_{B}$ is the number of ATHIS bands.

### 3.2. Spatial Noise Detection

Spatial noise detection is mainly to evaluate the noise status of each pixel. If the noise exceeds the threshold, it is judged as a blind pixel.

(1) To calculate the spatial distribution of the noise of the detector, we first calculated the noise of each detection unit itself. The calculation method is:
  (6) $$Noise_{bb}\left(i,k\right)=\sqrt{\frac{1}{N_{L,bb}}\sum_{j=1}^{N_{L,bb}}{\left[DN_{bb}\left(i,j,k\right)-DN_{bb}\left(i,k\right)\right]}^{2}}$$
(2) Then, we calculated the absolute deviation between the noise of each pixel in each band and the average noise of all the pixels in the band.
  (7) $$Noise_{bb,AD}\left(i,k\right)=\left|Noise_{bb}\left(i,k\right)-{\bar{Noise}}_{bb}\left(k\right)\right|$$
  where $Noise_{bb,AD}\left(i,k\right)$ is the absolute deviation of the pixel noise, and ${\bar{Noise}}_{bb}\left(k\right)$ is the average noise of all pixels in one band.
(3) The criterion of 3σ was used to extract the blind pixel mask on the $Noise_{bb,AD}\left(i,k\right)$. The mask was noted as $mask_{2}\left(i,k\right)$.
  (8) $$mask_{2}\left(i,k\right)=\left\{\begin{array}{c} 1\;\;\;\;Noise_{bb,AD}\left(i,k\right)>3\sigma_{bb,Noise}\left(k\right)\\ 0\;\;\;\;Noise_{bb,AD}\left(i,k\right)\leq 3\sigma_{bb,Noise}\left(k\right) \end{array}\right.$$
  where $\sigma_{bb,Noise}\left(k\right)$ is the root mean square of the *k*-th band in the absolute deviation data. The $\sigma_{bb,Noise}\left(k\right)$ could be calculated as
  (9) $$\sigma_{bb,Noise}\left(k\right)=\sqrt{\frac{1}{N_{S}}\sum_{i=1}^{N_{S}}Noise_{bb,AD}^{2}\left(i,k\right)}$$
  where $\;N_{S}$ is column number of ATHIS, representing the pixel number in the spatial dimension.

### 3.3. Response Slope Detection

Dark current pixels often have low response rates or negative response. Therefore, the pixel response slope could be used to detect the dark current pixels. The response slope detection mainly includes the following steps.

(1) To eliminate the drift of pixel values caused by the changes in the background radiation of the instrument, the pixels in the ATHIS background monitoring area shown in Figure 1 were selected, that is, the five bands with stable spectral responses. The warm and cold black body data are corrected based on the average value of all frames of the cold black body data.
  (10) $$DN_{wbb,corr}\left(i,k\right)=DN_{wbb}\left(i,k\right)+\frac{1}{5N_{S}}\sum_{k=246}^{250}\sum_{i=1}^{N_{S}}DN_{cbb}\left(i,k\right)\;-\frac{1}{5N_{S}}\sum_{k=246}^{250}\sum_{i=1}^{N_{S}}DN_{wbb}\left(i,k\right)$$
  where $DN_{wbb,corr}\left(i,k\right)$ is the 2D data of the warm black body after dark background correction.
(2) The response slope of each detector unit could be calculated according to the black body data after the process of (10).
  (11) $$Slope\left(i,k\right)=regress\left[X\left(i,k\right),Y\left(i,k\right)\right]$$

For the airborne calibration black body, the X(i, k) and Y(i, k) were

(12) $$X\left(i,k\right)=\left[DN_{cbb}\left(i,k\right),DN_{wbb,corr}\left(i,k\right)\right]\;Y\left(i,k\right)=\left[L_{cbb}\left(k\right),L_{wbb}\left(k\right)\right]$$

where $Slope\left(i,k\right)$ is the response slope of the pixel at the i-th column and the k-th band, $regress\left(\cdot \right)$ is the regression function, $X\left(i,k\right)$ is the vector of the pixel values of the black body after dark background correction, and $Y\left(i,k\right)$ is the vector of black body radiance corresponding to $X\left(i,k\right)$.

(3) The third mask noted as $mask_{3}\left(i,k\right)$ could be extracted as follows:
  (13) $$mask_{3}\left(i,k\right)=\left\{\begin{array}{c} 1\;\;\;Slope\left(i,k\right)<0\\ 0\;\;\;other \end{array}\right.$$

### 3.4. Blind Pixel Repairing in First Stage

After the aforementioned detection, three blind pixel masks were obtained. The union of these three masks was taken to get the final blind pixel mask, which was noted as $mask\left(i,k\right)$.

According to $mask\left(i,k\right)$, the average of the non-blind pixels in the 3 × 3 neighborhood of each abnormal pixel was selected as the value of the abnormal pixel. If there were no non-anomalous pixels in the 3 × 3 neighborhood $A_{w}$, the search window was expanded until the average value of the non-blind pixels could be obtained.

(14) $$DN_{bb,rep1}\left(i,k\right)=mean\left[DN_{bb}\left(i^{\prime },k^{\prime }\right)\right]\;\;\;i^{\prime },k^{\prime }\in A_{w},mask\left(i,k\right)=1$$

The coefficients of radiometric calibration could be calculated using the black body data after blind pixel repairing as:

(15) $$\left[a\left(i,k\right),b\left(i,k\right)\right]=regress\left[X_{rep1}\left(i,k\right),B\left(k\right)\right]$$

where $a\left(i,k\right)$ and $b\left(i,k\right)$ are the slope and intercept of the radiometric calibration function.

(16) $$X_{rep1}\left(i,k\right)=\left[DN_{cbb,rep1}\left(i,k\right),DN_{wbb,rep1}\left(i,k\right)\right]$$

(17) $$B\left(k\right)=\left[B_{cbb}\left(k\right),B_{wbb}\left(k\right)\right]$$

$B\left(k\right)$ is the warm and cold black body’s radiance in the corresponding band, and it can be calculated according to the black body temperature and spectral response function.

The blind pixels of the push-broom imaging detector always formed stripe noise on a specific band of aerial data. According to $mask\left(i,k\right)$, a method similar to (14) was used to repair the pixels of the corresponding band and position in the ATHIS image to obtain the repaired aerial image data $DN_{img,rep1}\left(i,j,k\right)$. In this way, the dead pixels, noise pixels, and dark current pixels in the imaging data with relatively stable response characteristics were repaired.

### 3.5. The Second Stage

The response characteristics of the blinking pixels were unstable, and it was difficult to detect all of them using warm-cold black body data. Based on $DN_{img,rep1}\left(i,j,k\right)$, we used the spectral filtering method to detect the abnormal bands in the spectrum of each pixel caused by the blinking pixels frame by frame. The linear interpolation was used to repair the values of the abnormal bands. The second stage was completed, and the final repair data $DN_{img,rep2}\left(i,j,k\right)$ were obtained.

## 4. Results and Discussion

### 4.1. Results of First Stage for Blind Pixel Detection

**Figure 4:**
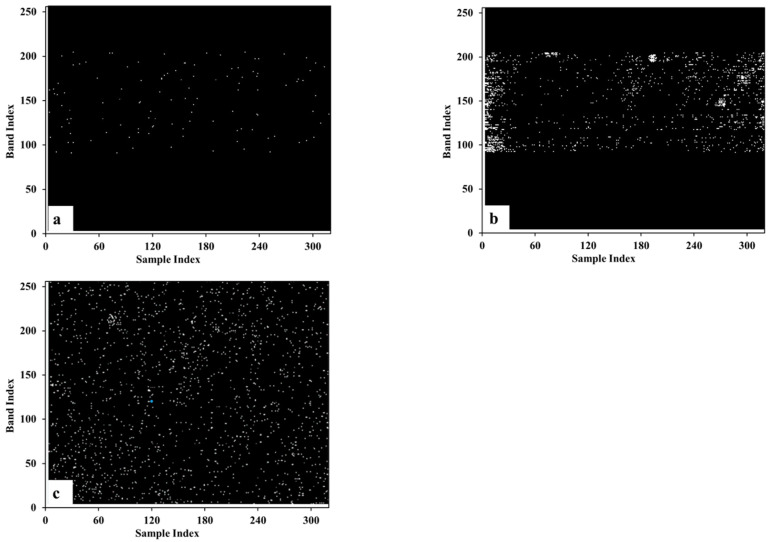
The extracted mask from black body data in laboratory using different methods. (**a**). GB/T 17444-2013; (**b**). zhang-2020; (**c**). Our method.

**Figure 5:**
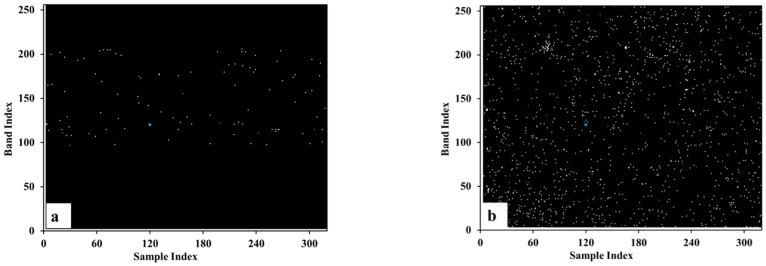
The extracted mask from on-board black body data using different methods. (**a**). GB/T 17444-2013; (**b**). our method.

It is not feasible to verify the results of blind pixel detection with simulated data. However, it is difficult to know the number and location of blind pixels in advance from measured data. Therefore, we mainly compare and analyze the distribution characteristics and quantity of blind pixels in the mask. Figure 4 and Figure 5 are the blind pixel masks extracted from the data of calibration black body in the laboratory and in the flight test, respectively, by different methods. It can be seen that the methods of the national standard (GB/T 17444-2013) and Zhang-2020 [12] can only detect blind pixels in the imaging area, but cannot detect blind pixels in the dark background monitoring area. Our method in this paper can perform blind pixel detection on two regions at the same time. In addition, the Zhang-2020 method requires more than three black body data at different temperatures so it cannot be used for blind pixel detection on on-board black body. From the perspective of the number of detected blind pixels, it is different on each data. On the one hand, it is due to the change of the blinking pixel; on the other hand, the threshold of the blind pixel detection algorithm is calculated based on actual data, so the threshold calculation results of different data are different, which also leads to the difference in the number of blind pixels. From the perspective of the distribution and number of blind pixels, the results of GB/T 17444-2013 and our method conform to the characteristics of random scattered distribution. The clustered distribution of blind pixels detected by the Zhang-2020 method does not meet the characteristics of blind pixel distribution, and there is a false alarm rate. The number of blind pixels detected by GB/T 17444-2013 is relatively small, only over 100 (Table 1). The comprehensive analysis shows that the blind pixel types detected by GB/T 17444-2013 are mainly dead pixels and noise pixels, and a small part is dark current and blinking pixels. The Zhang-2020 method can detect dead pixel, noise pixel, dark current pixel, and a few blinking pixels. The proposed method in this paper detects dead pixel, noise pixel, dark current pixel, and most blinking pixels.

**Figure 6:**
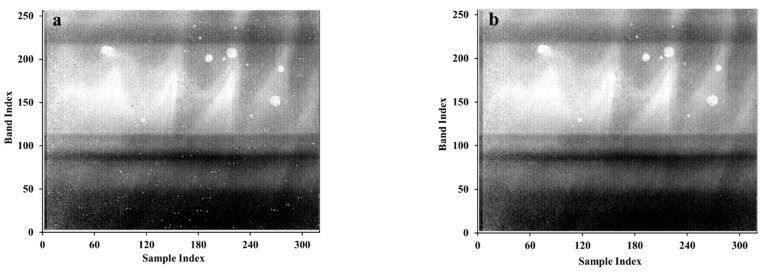
On-board black body data cube before (**a**) and after (**b**) blind pixel repair.

After processing by our method, almost all the blind pixels in the data cube of on-board black body have been repaired (Figure 6). Not only the blind pixels in the imaging region but also those in the background monitoring region have been well repaired.

### 4.2. Results of the Second Stage for Blind Pixel Dection

**Figure 7:**
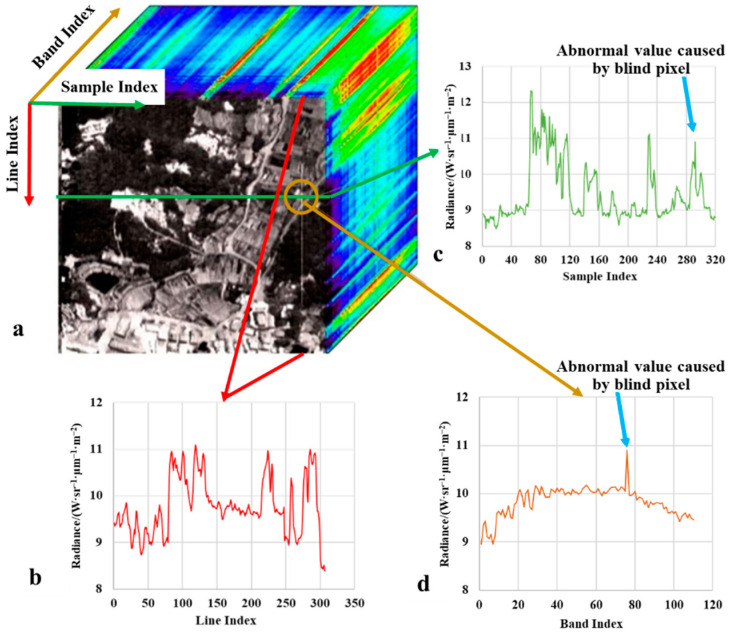
Thermal infrared hyperspectral data cube before blinking pixel repair. (**a**) data cube. (**b**) profile of along the line. (**c**) profile of along the sample. (**d**) spectrum of a specific pixel.

**Figure 8:**
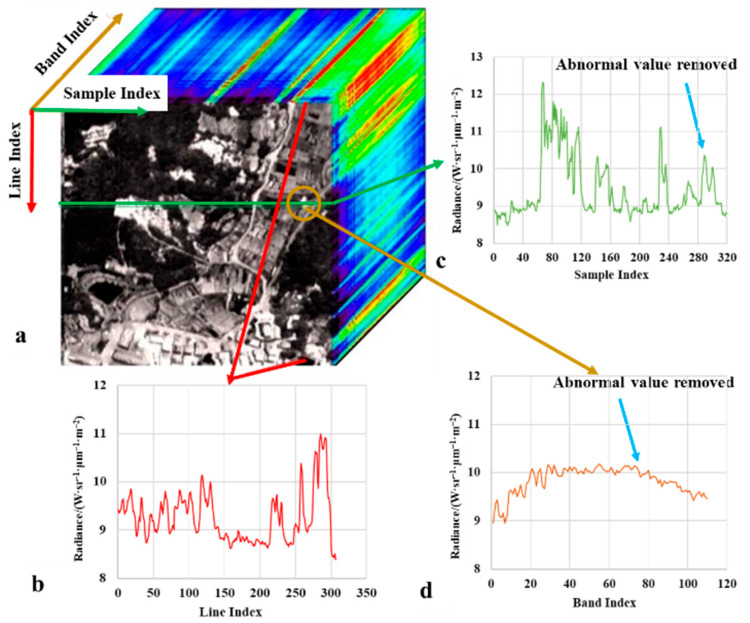
Thermal infrared hyperspectral data cube after blinking pixel repair. (**a**) data cube. (**b**) profile of along the line. (**c**) profile of along the sample. (**d**) spectrum of a specific pixel.

Figure 7 and Figure 8 are the thermal infrared hyperspectral data cubes obtained by ATHIS before and after the blind cell repair. The blinking pixel usually appears as an uninterrupted obvious line on the image, such as the bright line in Figure 7a. This is mainly due to the intermittent response of the blinking pixel, which causes the value of the pixel in the image of some frames to be normal, while the value of the pixel in other frames is abnormal. Therefore, in the process of detecting and repairing the spectral abnormality caused by the blinking pixel, the response value of the blinking pixel in all frames should not be arbitrarily regarded as abnormal, but the original information can be retained as much as possible by analyzing frame by frame. Comparing the profile of line and sample (Figure 7b,c) and the spectrum (Figure 7d) of the pixel, it can be seen that the abnormal value of the pixel caused by the blinking pixel is difficult to be identified in the spatial dimension, but easier to be identified in the spectral dimension. The spectral filtering method we use achieves this goal. After the processing of the proposed method, the sudden change of pixel value starting from the Line 5250 is corrected (Figure 7b and Figure 8b). The bright line caused by the detector’s blinking unit is removed, that is, the abnormal value of the pixel in each frame is repaired (Figure 7a and Figure 8a).

### 4.3. Inversion of Temperature and Emissivity

**Figure 9:**
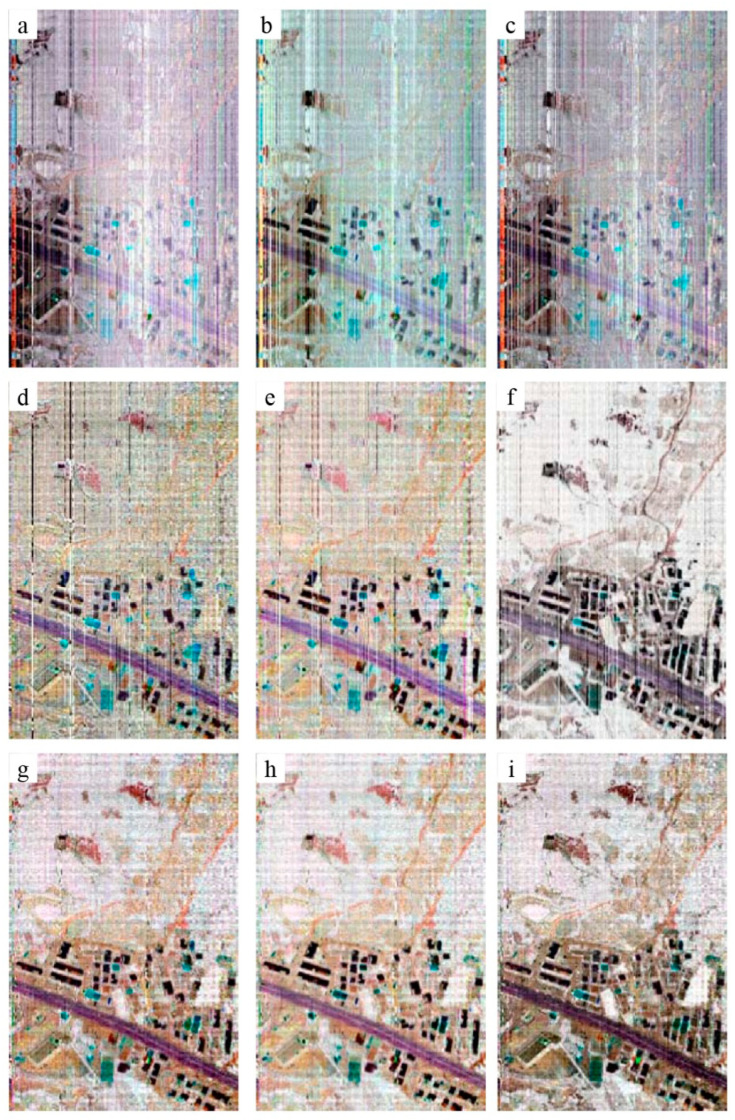
The emissivity retrieved by the commonly used TES algorithms based on the ATHIS data before (**a**–**c**) blind pixel repair, (**d**–**f**) blind pixel repair by GB/T 17444-2013 method and (**g**–**i**) blind pixel repair by proposed method. (**a**,**d**,**g**: ARTEMISS; **b**,**e**,**h**: WTTES; **c**,**f**,**i**: RDSS.).

**Figure 10:**
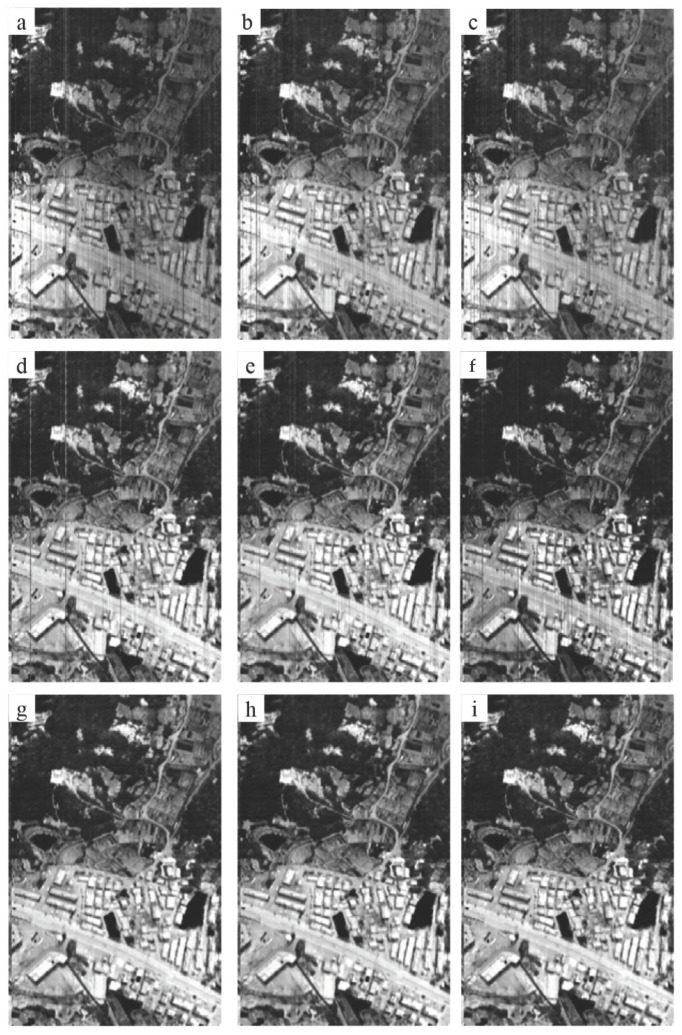
The temperature retrieved by the commonly used TES algorithms based on the ATHIS data before (**a**–**c**) blind pixel repair, (**d**–**f**) blind pixel repair by GB/T 17444-2013 method and (**g**–**i**) blind pixel repair by the proposed method. (**a**,**d**,**g**: ARTEMISS; **b**,**e**,**h**: WTTES; **c**,**f**,**i**: RDSS.).

Figure 9 and Figure 10 are the emissivity and temperature images obtained by inversion of different temperature and emissivity separation (TES) algorithms, respectively. Figure 9a–c and Figure 10a–c are the inversion results based on the data before the blind pixel repair. Figure 9d–i and Figure 10d–i are the results based on the data after blind pixel repair. We selected several TES algorithms with higher accuracy reported in recent years for comparison. There are mainly automatic retrieval of temperature and emissivity using the spectral smoothness method (ARTEMISS) [13], wavelet transform method for separating temperature and emissivity (WTTES) [14], and resolution-degrade-based spectral smoothness (RDSS) [15]. It can be seen from Figure 9 and Figure 10 that there are a large number of light and dark strips in the temperature and emissivity images obtained from the data before blind pixel repair. The Zhang-2020 method requires black body data at three different temperatures to perform blind pixel detection, but the airborne radiation calibration process only has black body data at two temperatures. Therefore, Zhang-2020 is not applicable for on-board data processing. By comparing the repaired results of GB/T 17444-2013 and our proposed method, we can see that there are still a certain number of strips in the emissivity and temperature images retrieved from the data repaired by GB/T 17444-2013 method. While, there are no strips in the result images obtained from the blind pixel repaired data by the proposed method (Figure 9 and Figure 10).

**Figure 11:**
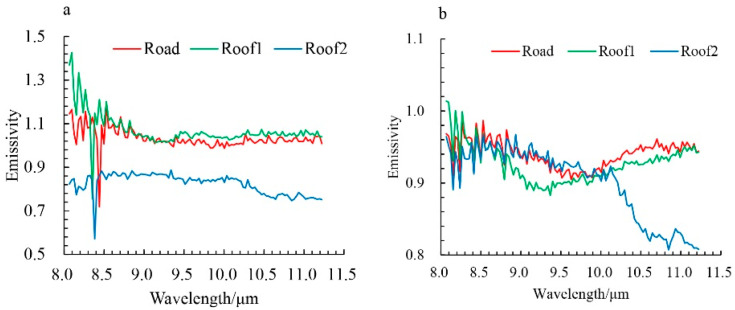
The retrieved emissivity spectrum based on the data before (**a**) and after (**b**) blind pixel repair.

The influence of the blind pixel on the inversion result is not only the strip noise on the image but also the peak of the emissivity spectrum and the overall deviation of the emissivity value in the spectral dimension. Figure 11 shows the emissivity spectrum of roads and roofs on the result image. The emissivity value deviates, and its corresponding temperature also deviates greatly from the true value (the emissivity is theoretically between 0 and 1). Although the spikes in Figure 11a can be removed by spectral filtering in the post-processing of the results, the deviation of emissivity cannot be corrected. From this point of view, under the current inversion technology, repairing blind pixel is crucial for the inversion of temperature and emissivity.

## 5. Conclusions

Considering the imaging principles and characteristics of ATHIS, we proposed a novel real-time method for blind pixel detection and repair. This method obtains the blind pixel mask by performing spectral filtering detection, spatial dimensional noise detection, and response slope detection on the calibration black body data cube and then repairs the blind pixels with stable characteristics, such as dark current noise and dead pixels. Then, the spectral filtering method is used to repair the blind pixels with large randomness, such as blinking pixels. We applied the proposed method to the thermal infrared hyperspectral data obtained in the field flight experiment to test the method. The experimental results show that the proposed method effectively removes the striped noise on the image caused by the blind pixels of the detector and significantly improves the image quality and the inversion accuracy of temperature and emissivity. This method can not only remove and repair the blind pixels of the data obtained by ATHIS but also has a very important reference value for the blind pixel removal of other push-broom thermal infrared hyperspectral imagers.

## Figures and Tables

**Table 1:** Number of detected blind pixel using different methods.

Method	Black Body in Laboratory	On-Board Black Body
GB/T 17444-2013	119	100
Zhang-2020	1534	—
Our method	1819 (736) ^a^	1404 (555) ^a^

^a^ 736 and 555 are the number of blind pixels in the imaging region.
